# Evaluation of deep learning-based approaches for COVID-19 classification based on chest X-ray images

**DOI:** 10.1007/s11760-020-01820-2

**Published:** 2021-01-07

**Authors:** Kamal KC, Zhendong Yin, Mingyang Wu, Zhilu Wu

**Affiliations:** School of Electronics and Information Engineering, Harbin Institute of Technology, Harbin, 150001 China

**Keywords:** Deep convolution neural network, Transfer learning, Chest X-ray, COVID-19, SARS

## Abstract

The COVID-19, novel coronavirus or SARS-Cov-2, has claimed hundreds of thousands of lives and affected millions of people all around the world with the number of deaths and infections growing exponentially. Deep convolutional neural network (DCNN) has been a huge milestone for image classification task including medical images. Transfer learning of state-of-the-art models have proven to be an efficient method of overcoming deficient data problem. In this paper, a thorough evaluation of eight pre-trained models is presented. Training, validating, and testing of these models were performed on chest X-ray (CXR) images belonging to five distinct classes, containing a total of 760 images. Fine-tuned models, pre-trained in ImageNet dataset, were computationally efficient and accurate. Fine-tuned DenseNet121 achieved a test accuracy of 98.69% and macro f1-score of 0.99 for four classes classification containing healthy, bacterial pneumonia, COVID-19, and viral pneumonia, and fine-tuned models achieved higher test accuracy for three-class classification containing healthy, COVID-19, and SARS images. The experimental results show that only 62% of total parameters were retrained to achieve such accuracy.

## Introduction

COVID-19 , novel coronavirus or SARS-CoV-2, is a human coronavirus within the virus family Coronaviridae, order Nidovirales, which are multi-lipid, enveloped non-segmented, single-strained, positive-sense RNA viruses named after corona or crown-like surface projections [1]. In December 2019, Wuhan, the capital of central China’s Hubei province, became ground zero for COVID-19 outbreak. As of June 2020, more than 10 million cases of infections and 500 thousands deaths have been reported from more than 213 countries and territories. Severe acute respiratory syndrome (SARS) and Middle East respiratory syndrome (MERS), the predecessor to COVID-19, are viral respiratory diseases. SARS-Cov first reported in November 2002 in Guangdong province of southern China, spread to 29 countries with more than 8000 infected cases and 774 deaths. MERS-Cov was first reported in Saudi Arabia in September 2012 and has spread to 27 countries with more than 2500 infections and 866 deaths. In general, respiratory virus infection can occur through direct or indirect contact, droplet spray in short-range transmission and aerosol in long-range transmission [2].

COVID-19 patients develop common symptoms like fever, dry cough, myalgia, fatigue, dyspnea, and anorexia at onset of illness. In due course of time, these symptoms progress to acute respiratory distress syndrome (ARDS), arrhythmia, and shock [3]. X-rays are the most common, cheap, and widely available diagnostic imaging technique used to detect and diagnosis tumors and other abnormal masses like lymph nodes, pneumonia, bone fractures, abscesses, etc. X-rays reveal abnormalities indicative of lung diseases. Early stages of CXR radiograph of COVID-19 may read as normal, but with progression in time, the readings may resemble of pneumonia or ARDS. It is a difficult task to classify these X-ray images between normal, bacterial pneumonia, viral pneumonia, SARS or COVID-19 irrespective of community acquired pneumonia (CAP) or hospital-acquired pneumonia (HAP). Chest X-rays can be used as preliminary classification due to its prevalent usage as primary diagnostic test.

AI researchers have contributed a lot in the field of data processing. Advances in AI have the potential in transforming the field of medicine since medical diagnostics and treatments are fundamentally a data problem. Deep learning, a sub-field of machine learning, automatically extracts detailed features from data which has proven to be highly efficient in various data processing task. Deep learning has become a prominent solution for a wide variety of natural language processing (NLP) or text processing, speech recognition and synthesis, signal analysis, and computer vision tasks. CNNs, multilayered neural networks, play a pivotal role in various digital image processing tasks such as feature extraction, pattern recognition, segmentation, object recognition, classification on various types of images including medical images. Deep learning demands plethora of data to achieve acceptable result and is resource hungry. To overcome this difficulty, a machine learning technique, transfer learning, was introduced. Transfer learning re-purposes a model trained on one task on a second related task. Pre-trained models are easily re-trained on smaller data with lesser trainable parameters to achieve higher accuracy. Depending upon the data and model selected, pre-trained models are fine-tuned to attain fine result.

## Related works

Several machine learning algorithms have been used for automatic classification of digitized medical images. Machine learning recognizes patterns to compute image features suitable for detection, diagnosis, or classification tasks. Machine learning algorithms can be classified on the basis of training styles: supervised, unsupervised, and reinforcement learning. Supervised learning involves gaining experience by using images that contain useful information and applying the gained expertise to predict on unseen new images (test data). Examples of supervised learning algorithms include support vector machine (SVM), decision tree, linear regression, logistic regression, naive Bayes, k-nearest neighbor (k-NN), random forest, AdaBoost, and neural network methods. In unsupervised learning, data are processed with a goal of separating the images into groups. Since the algorithm does not have information regarding the groups, it automatically determines the number of groups and a way to separate them. Examples of unsupervised learning algorithm systems include K-means, hierarchical clustering, density-based spatial clustering of applications with noise (DBSCAN), Markov random fields (MRF), iterative self-organizing data (ISODATA), and fuzzy C-means (FCM) systems. Like supervised learning, reinforcement learning begins with a classifier that was built by using labelled data. However, the system is then given unlabelled data, and it tries to further improve the classification by better characterizing these data similar to unsupervised learning. Examples of reinforcement learning algorithm systems include Monte Carlo methods and temporal difference (TD) algorithms [4]. Several machine learning or deep learning algorithms have been used for automatic classification of medical images based on X-rays [5], CT scans [6], mammograms [7], MRI [8], PET scans [9], or RGB images [10] for detection of tumor, diabetic retinopathy (DR), cancer, Alzheimer’s disease, pneumonia, or a RGB image for skin lesion classification.

### Deep learning in medical images

Deep learning is a new and powerful data oriented machine learning method, which utilizes a range of neural network architectures to perform various imaging tasks, like object (i.e., lesion or region of interest) detection, segmentation and classification. Deep learning methods differ from the conventional machine learning methods (i.e., support vector machine (SVM), k-NN, naive Bayes, and random forest, etc.) in one major sense: The latter rely on feature extraction methods to train the algorithm, whereas deep learning methods extract feature automatically to learn the image. Evaluated on the 150 validation images from the ISIC 2017 classification challenge for skin lesion classification, a fusion of three pre-trained deep models, AlexNet, VGG16, and ResNet-18, as deep feature generators yielded an area under receiver operating characteristic (ROC) curve of 83.83% for melanoma classification and of 97.55% for seborrheic keratosis classification [11]. Recurrent Attention DenseNet (RADnet) which employs original DenseNet architecture along with adding the components of attention for slice level predictions and recurrent neural network layer for incorporating 3D context was employed for classification of dataset composed of 185, 67, and 77 brain CT scans for training, validation, and testing, respectively. RADnet achieved 81.82% hemorrhage prediction accuracy and 84.78% F1 score [12].

### Convolutional neural network for pneumonia and COVID-19 detection

A four convolutional layered CNN was employed for binary classification of presence of pneumonia based on a total of 5856 X-ray images of anterior–posterior chests from retrospective pediatric patients of which 3722 images were allocated to the training set and 2134 images to the validation set. The images were augmented using various techniques like rescale, rotation, shift, shear, zoom, horizontal ip, etc. The network achieved a validation accuracy of 93.73% on input data of size $200 \times 200$ [13]. An ensemble model based on transfer learning reached an accuracy of 96.4% with a recall of 99.62% on unseen data from the Guangzhou Women and Children’s Medical Center dataset for binary classification of healthy and pneumonia X-Ray images [14]. Classification of 50 images of healthy and 50 images of COVID-19 patients using pre-trained ResNet50 provided the classification performance of 98% accuracy [15]. Deep learning with X-ray imaging may extract significant biomarkers related to the COVID-19 disease was exhibited by training and testing on an unbalanced dataset of 1442 X-ray images including 224 images with confirmed COVID-19 disease, 714 cases of both bacterial and viral pneumonia (400 bacteria and 314 viral), and 504 images of normal conditions. MobileNetV2 outperformed other models and obtained 94.72% accuracy [16]. COVID-Net network architecture which uses a lightweight residual projection-expansion projection-extension (PEPX) design pattern was used on a dataset which comprised of 183 cases of COVID-19, 5538 of Pneumonia and 8066 of healthy subjects with a test set of 100 images of pneumonia and healthy lungs, and 31 of COVID-19 with no patient overlap between the test and the training set. The model achieved a test accuracy of 93.3% [17]. Covidx-Net used transfer learned models to classify healthy and COVID-19 cases and concluded that VGG19 and Densenet201 achieved highest accuracy of 90% [18]. A total of 250 COVID-19 and 250 non-COVID-19 images for training, and an independent test set of 74 positive and 36 negative samples were trained with an ensemble of ten CNNs and achieved an AUC of 0.81 for the classification task [19].

## Materials and methods

### Proposed approach

In this work, we employ data-driven deep learning technique, convolutional neural network (CNN). Since its introduction by Lecun and due to its unrivalled performance in 2012 ImageNet challenge achieved by AlexNet, CNN has become a prominent technique in image processing tasks. Deep learning or data-driven approaches, in general, require abundance of data to generalize from training data and discover viable feature sets and decision criteria, and finally extract meaningful results. Various state-of-the-art deep learning models such as VGG-16, and VGG-19 [20], Inception [21], ResNet [22] , Xception [23], which performed well in ILSVRC [24] make use of CNN and its variant. We propose fine-tuning of a transfer learned model trained on ImageNet dataset.

#### VGG

In 2014, Oxford’s Visual Geometry Group (VGG) developed and trained a 16 convolutional layered deep convolutional network for object recognition called VGGNet. Different versions of VGGNet were introduced like VGG11, VGG11 (LRN), VGG13, VGG16 (Conv1), VGG16,, and VGG19. VGG19 is 19 layers deep with each layer using a filter of size $3\times 3$ and has an image input size of $224\times 224$.

#### InceptionV3

**Fig. 1:**
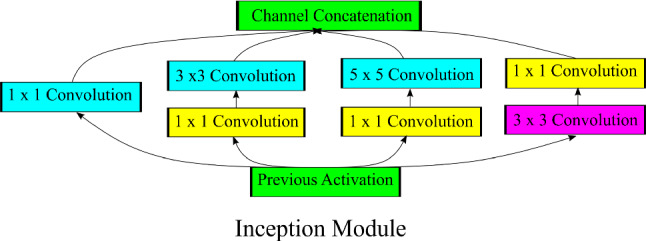
Inception module: building block of inception model

Szegedy et al. [21] proposed inception module in GoogleNet or InceptionV1 that was designed to be efficient in terms of parameters and computations, while achieving state-of-the-art performance. Different versions of Inception model were introduced by optimizing inception module using Batch Normalization, factorized convolution and residual connections. InceptionV3 is a 42 layered deep network. Figure 1 shows the inception module which is the building block of inception model.

#### ResNet

At the ILSVRC 2015, Residual Neural Network (ResNet) by He et al. [22] introduced a novel architecture with residual connections which greatly reduced the optimization difficulties and enabled the training of much deeper networks by introducing skip connections between residual modules.

**Fig. 2:**
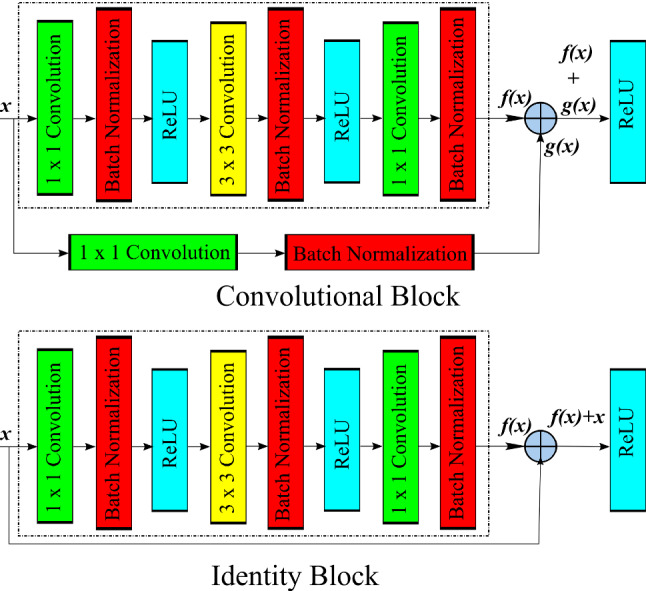
Convolutional block and identity block which combine to form residual network

ResNet50 is a 50-layer deep neural network. Figure 2 shows convolutional block and identity block which combine to form residual network.

#### MobileNet

The need for smaller model size suitable for real-time analysis in resource constrained mobile devices led to the development of MobileNet [25], which uses depthwise separable convolutions as their core building blocks. MobileNets are small, low-latency, low-power models parameterized to meet the resource constraints of a variety of use cases by introducing width and depth multiplier used for classification, detection, embeddings and segmentation.

#### DenseNet

**Fig. 3:**
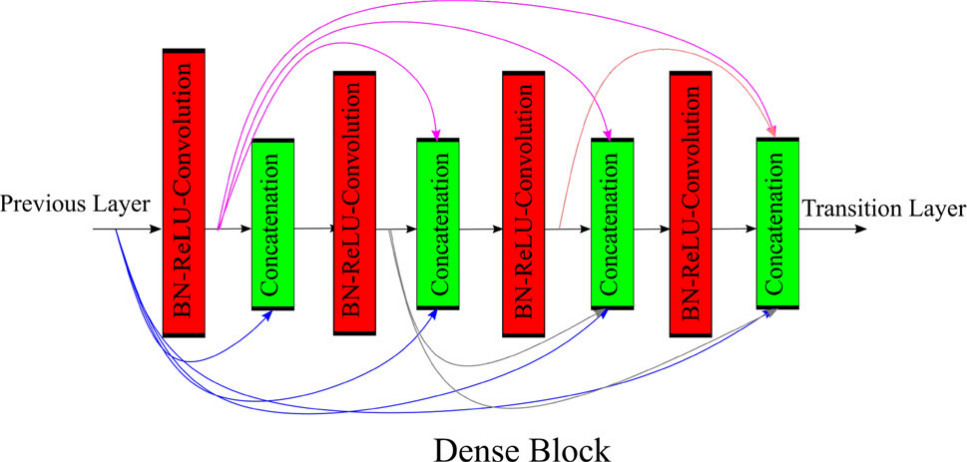
Dense block: basic building blocks for DenseNet

Dense blocks were firstly proposed by Huang et al. in DenseNet [26] that introduced dense connectivity in which each layer receives signals from all its preceding layers combined by channel-wise concatenation resulting into low information bottleneck. DenseNets integrate the properties of identity mappings, deep supervision, reduced feature redundancy and diversified depth allowing feature reuse making it good feature extractors. DenseNets with different depths; DenseNet-121,169,209,264 were introduced. Figure 3 shows dense block, the basic building block used in DenseNet.

**Table 1:** Chest X-ray dataset split statistics

Class	CXR A			CXR B			CXR C		
	Train	Val	Test	Train	Val	Test	Train	Val	Test
Bacterial Pneumonia	134	33	19	118	30	38	–	–	–
COVID-19	134	33	19	118	30	38	118	30	38
Normal	134	33	19	118	30	38	118	30	38
Viral Pneumonia	134	33	19	118	30	38	–	–	–
SARS	–	–	–	–	–	–	10	3	3

#### NasNetMobile

Google Brain designed a search space, called NASNet search space, which decouples the complexity of an architecture from the depth of the network, thus resulting in transferable and scalable network called Neural Architecture Search Network (NASNet). NASNet comprises of two types of cells: normal cell that returns a feature map of the same dimension and reduced cell that returns a feature map where the feature map height and width is reduced by a factor. NASNetMobile [27], also referred as NASNet-A(4 @ 1056) is a reduced version of NASNetLarge, referred as NASNet-A(6 @ 4032).

### Transfer learning and fine-tuning

Transfer learning is a machine learning technique that involves reusing an existing, trained neural network, developed for one task, as the foundation for another task. Transfer learning removes the necessity of large training data for a task as the basic features required to train a model are imported from previous task. In DNN, each layer generate a sequence of representations of the data. For an image recognition network, the early layers in the CNN capture the simplest features of the data that are universal such as the presence of edges, contours, or gradients in color and can be reused for similar tasks, while later layers capture more complicated which are highly data dependent and needs to be re-trained. The main challenge of transfer learning is to retain the existing knowledge in the model while adapting the model to new task which gives rise to the problem of the number of layers or parameters required to be re-trained to achieve optimal result.

Transfer learning involves firstly finding the suitable pre-trained model, secondly, replacing the final layer of the model according to the number of output layers for the new task and finally, resume training the model with new data and fine-tuning the model till the accuracy converges towards higher value.

## Methodology

Deep neural network is a stack of layers where each hidden layer extracts meaningful features automatically. This feature of DNNs is utilized in transfer learning. Transfer learning is employed whenever the amount of training data is smaller in number. Transfer learning is done either by training a model from scratch on a suitable huge dataset and retraining the model on desired smaller dataset or using a pre-trained state-of-the-art models. The former exploits lot of resources and time as designing a model, creating a huge dataset and then finally training them is tiresome. However, using state-of-the-art model pre-trained on standard dataset reduces lots of computational cost.

**Fig. 4:**
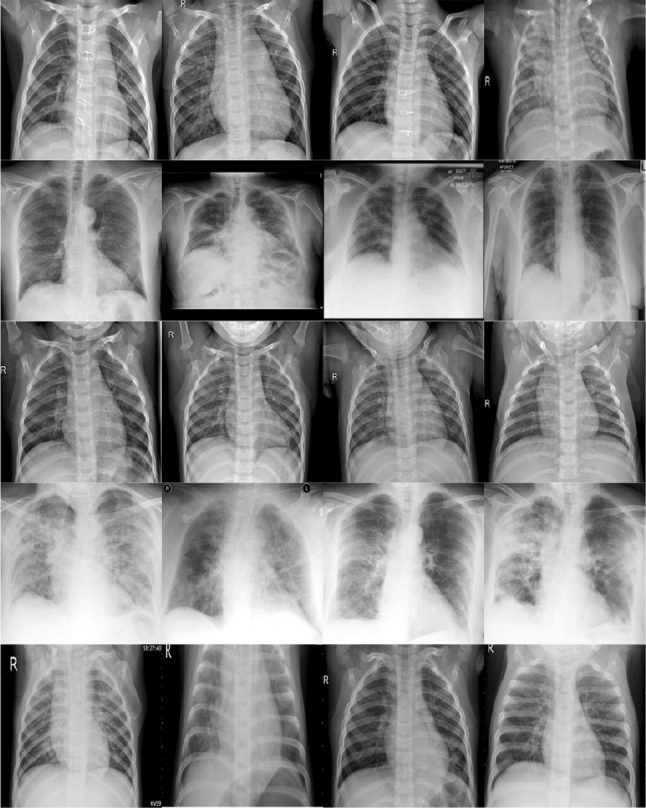
Some samples from our dataset which includes an image from each class. Each row represents x-ray images from each class. From top row to bottom: bacterial pneumonia, COVID-19, normal, SARS and viral pneumonia

Since collecting large amount of medical images is difficult due to data privacy concerns, transfer learning is opted as a suitable solution. Transfer learning makes use of reusable features by using the concept of layer freeze. Since each layer outputs some features in DNN, spotting the layer best suited for extracting the reusable feature is delicate. Freezing a certain portion of network that best provides the features which can be used in new domain is the basic principle of transfer learning. However, employing transfer learning on models pre-trained in ImageNet demands the replacement of final output layer with the new layer suited for the new task. The network can be either trained by freezing all the layers except the final layers which is termed as feature extractor or freezing a portion of network for best results which is termed as fine-tuning. We opted for fine-tuning instead of feature extractor for higher accuracy in expense of slightly higher computational cost [28]. Fine-tuning is conducted by using the concept of block freeze in which a collection of fixed number of layers are bundled to form a block. This collection of blocks are frozen instead of layers. Parameters corresponding to the frozen layers are referred as non-trainable parameters, whereas the network trains with remaining trainable parameters. Freezing the layers does not affect the weights corresponding to the layer. All the experiments were run for 1000 epochs.

## Dataset

The dataset used is a collection of two subsets from two different dataset, COVID-19 dataset [29] created by Dr. Joseph Cohen and Pneumonia dataset [30] which are Chext X-Ray dataset. The dataset was divided in two ways. In the first scenario, CXR A, the dataset was divided into training and testing set with 90:10 split, and the training set was further split into training and validation set with 80:20 split, respectively. In the second scenario, CXR B, the dataset was divided into training and testing set with 80:20 split, and the training set was again split into training and validation set with 80:20 split, respectively.

The chest X-Ray contains images collected from various patients all around the world. A total of 744 images, equally split over four classes (normal, bacterial pneumonia, viral pneumonia, and COVID-19), were used for classification. The CXR C contains a total of 388 X-ray images from three different classes (healthy, COVID-19, and SARS). Data are split similar to second scenario. While CXR A and B have balanced data, data in CXR C are highly imbalanced. The imageset contains AP (anterior–posterior) and PA (posterior–anterior) CXR images. L (Lateral) X-ray images are discarded for the experiment. These x-rays were taken either erect (standing or sitting) or supine position. Few of the X-rays contain images from patients with chest port inserted in them. While most of the chest X-ray images were taken from different patients all around the world, COVID-19 dataset contain some X-rays from a single patient over a course of time, i.e., x-rays from day 1, day 8 and day 13.

A detailed information about the images is shown in Table 1. Images are either in jpg, jpeg, or png format. Figure 4 shows few samples of images used in the experiment. Images from each classes are shown in the figure.

**Table 2:** Comparing validation and test accuracies (%) on fine-tuned models on chest X-ray dataset

Models	CXR A		CXR B	
	Validation	Test	Validation	Test
VGG19	**100**	97.37	**100**	94.74
InceptionV3	**100**	97.37	**100**	97.37
ResNet50	**100**	97.37	**100**	94.08
ResNet50V2	**100**	97.37	**100**	94.08
MobileNet	**100**	97.37	**100**	95.4
MobileNetV2	**100**	97.37	**100**	95.4
DenseNet121	**100**	97.37	**100**	**98.69**
NasNetMobile	**100**	97.37	98.44	97.37

Bold denotes the highest value for particular category

**Fig. 5:**
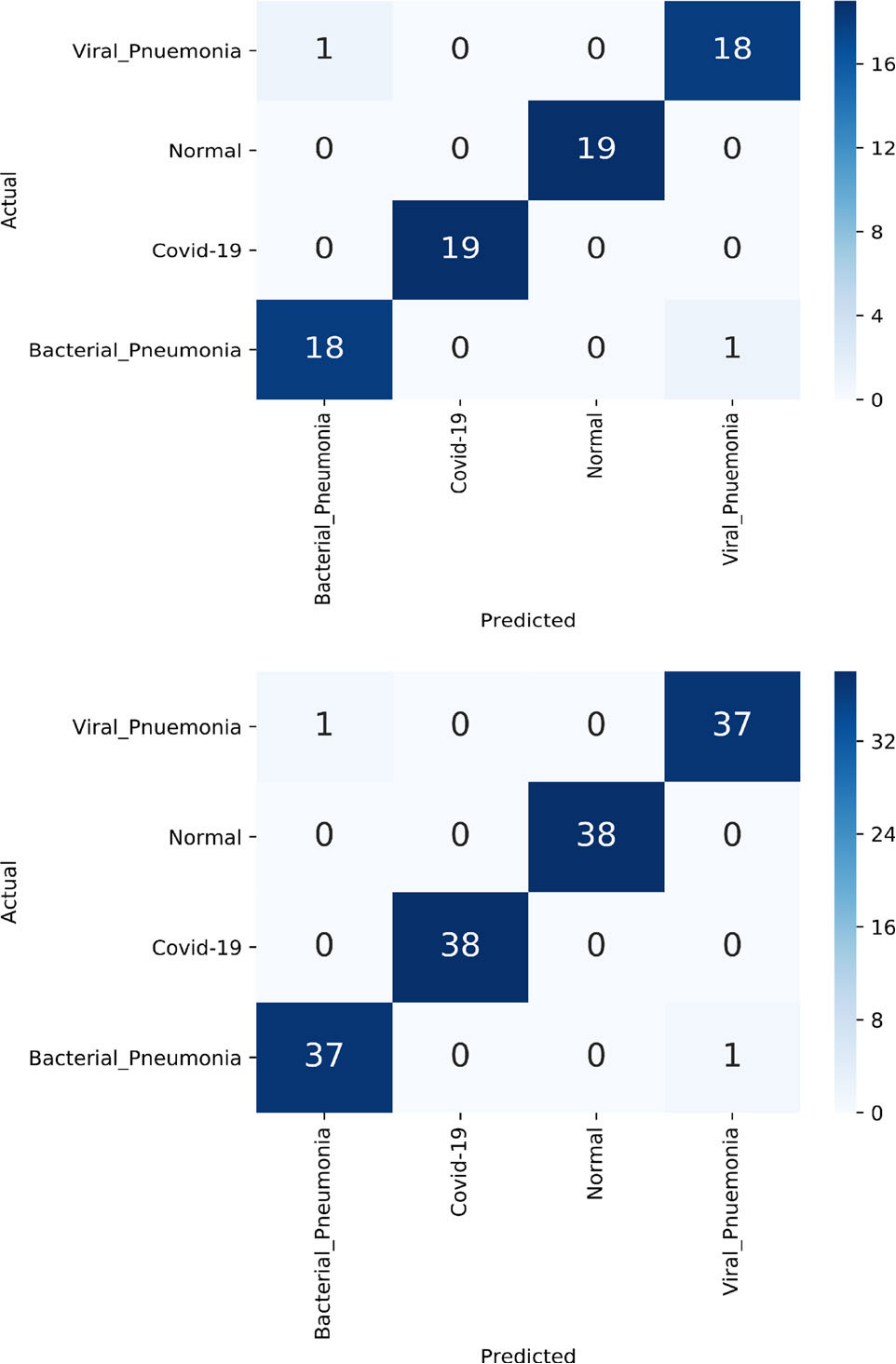
Confusion matrix for CXR dataset. Confusion matrix on the top depicts prediction result of CXR A dataset and the one in the bottom depicts that of CXR B dataset

## Results

Transfer learned eight different state-of-the-art models pre-trained on ImageNet achieved 97.37% test accuracy on CXR A dataset. Fine-tuned DenseNet121 outperformed other models by achieving 98.69% test accuracy on CXR B dataset. Table 2 shows the accuracies obtained while employing fine-tuned models on CXR dataset. All the models achieved a validation accuracy of 100% except for NasNetMobile on CXR B dataset. Data split of 80:20 (train/test) with 80% further into 80:20 (train:validation) split performed better compared to 90:10 (train:test) with 90% further into 80:20 (train/validation) split. The confusion matrix of CXR A and CXR B for test data is given in Fig. 5.

**Fig. 6:**
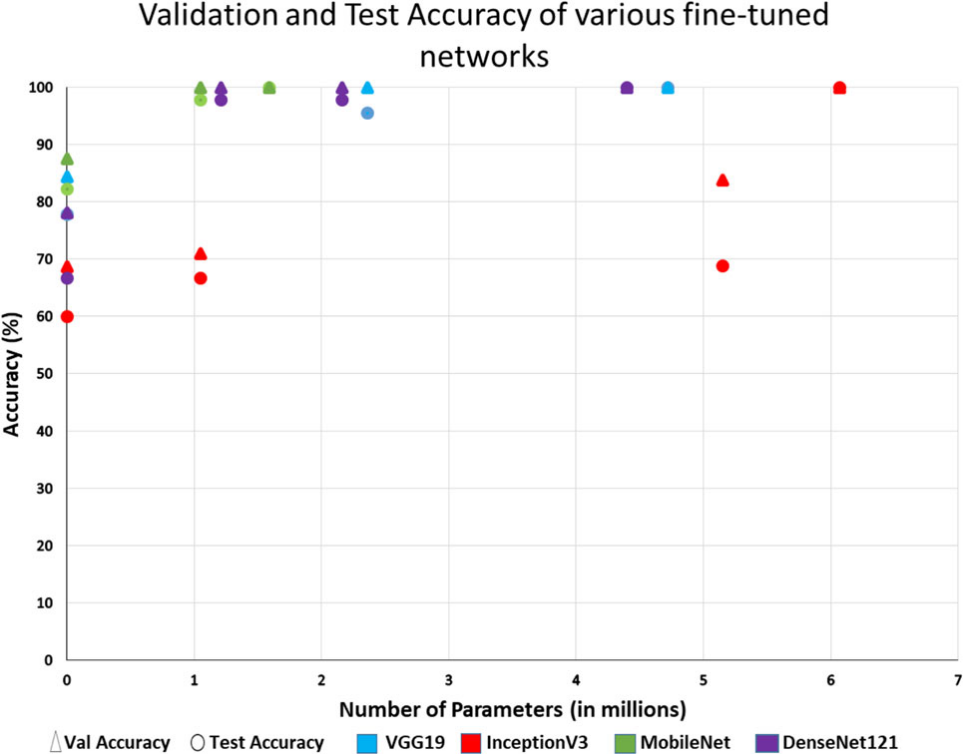
Validation and test accuracies attained by various models in CXR C dataset during fine-tuning. Triangles and circles represent validation and test accuracy, while different colors represent different networks

Table 2 shows the comparison of validation and testing accuracy of CXR A and CXR B obtained after fine-tuning various standard pre-trained models. Table 3 shows the classification performance measure of CXR datasets. CXR B obtained the highest macro f1-score of 0.99 when fine-tuned using DenseNet121. Table 4 compares accuracy of various existing models and our model. Table 5 shows the respective number of layers frozen, trainable parameters, and the percentage of total parameters trained by various fine-tuned models to obtain highest accuracy. The scatter plot in Fig. 6 shows the validation and testing accuracy of various fine-tuned models. While employing transfer learning using block freeze, accuracies depend on the number of trainable parameters. It can be seen that accuracy increases as number of parameters increase.

**Table 3:** Classification performance measures on chest X-ray dataset

Dataset	Class	Precision	Recall	F1-score	Macro F1-score
CXR A	Bacterial	0.95	0.95	0.95	0.975
	Covid19	1	1	1	
	Normal	1	1	1	
	Bacterial	0.95	0.95	0.95	
CXR B	Bacterial	0.98	0.98	0.98	**0.99**
	Covid19	1	1	1	
	Normal	1	1	1	
	Bacterial	0.98	0.98	0.98	

Bold denotes the highest value for particular category

## Conclusion

In this work, various fine-tuned state-of-the-art deep learning models pre-trained on ImageNet, were compared for efficient classification of chest X-ray images. These models effectively classified healthy, bacterial, viral, COVID-19 and SARS diseases based on CXR images. Fine-tuned models achieved validation accuracy of 100% on all CXR dataset, 100% test accuracy on CXR C, 97.37% of test accuracy on CXR A dataset and 98.69% of test accuracy on CXR B dataset by fine-tuned DenseNet121. DenseNet121 outperforms all models in terms of precision, recall, f1-score, and accuracy, thus, it is proven to be the most effective model for the specific classification task, and the specific data sample.

**Table 4:** Comparison with existing models

Models	Accuracy (%)	# images (# classes)	Imaging type
Covid-Net [17]	93.3	13972 (3)	X-ray
Transfer [16]	96.78	1428 (2)	X-ray
Learning			
De-Trac [31]	95.12	196 (3)	X-ray
DTL + [32]	96	2492 (2)	CT
DenseNet201			
DeConvNet [33]	90.1	630 (2)	CT
GSA- [34]	98	414 (2)	X-ray
DenseNet121			
DarkNet [35]	98.08	527 (2)	X-ray
Our method	**98.69**	744 (4)	X-ray

Bold denotes the highest value for particular category

**Table 5:** Number of frozen layers and trainable parameters for CXR C

Models	Number of layers frozen	Trainable parameters (M)	% of total parameters
VGG19	19	4.72	23.6
InceptionV3	279	6.073	27.9
MobileNet	75	1.59	49.4
DenseNet121	224	4.4	62.5

All the models were pre-trained on ImageNet dataset. ImageNet demonstrated to be a reliable source domain for transferring learned features on CXR disease classification. The models performed well for classification of healthy and disease X-ray images of COVID-19, SARS diseases. Dataset CXR A and CXR B had equal number of images per class while CXR C dataset had unbalanced data with one class sparse amount of data. This signifies that transfer learning method works well with unbalanced and small dataset.

Using the concept of block freeze instead of layer, reduced the time required to pinpoint the layer that gives higher accuracy with less number of trainable parameters. Splitting the data into 80% for training and 20% for testing instead of 90% and 10% for training and testing respectively gives higher accuracy and f1 score. This study explored various deep learning models and transfer learning techniques by creating and comparing their efficacy on CXR images for COVID-19 classification, while keeping computational demands small. This model can be used as preliminary test for COVID-19 detection.
